# Elements of Physiology, Including Physiological Anatomy, for the Use of the Medical Student

**Published:** 1846-06

**Authors:** 


ART. XII.—*Elements of Physiology, including Physiological Anatomy, for the use of the Medical Student.* By WILLIAM B. CARPENTER, M.D., F.R.S., Fullerism Professor of Physiology in the Royal Institution of Great Britain; member of the American Philosophical Society; and Corresponding member of the National Institute of the United States, &c., &c. With 180 Illustrations. Philadelphia: Lea & Blanchard. 1846. 8vo. pp. 566.

Dr. Carpenter is the author of several works on Physiology which have been well received. His "Principles of Physiology," published some years ago, is in the hands of many of our readers; besides this elaborate work, he has written a "Popular Treatise on Vegetable Physiology," which Messrs. Lea & Blanchard have issued in this country; and another volume from his pen entitled "Principles of General and Comparative Physiology" is going through the press of the same publishers. The work before us is designed especially for students, as a manual to accompany them in the study of the elements of physiological science. It necessarily combines, in some degree, the scope of his two larger works, yet it has claims to be regarded as something more than a mere abridgment of them, the plan on which it is written being in many respects different. He commences his exposition of the characters of "organized structures and of vital phenomena by a full account of the development and metamorphoses of *cells*, and of the purposes which these effect in the living body, either in their original or their altered condition." Dr. Carpenter believes that the inferences, which may be drawn from the observations on this subject, "are entitled to hold the same rank in physiological science as that taken by the doctrine of mutual attraction in general physics, or of elective affinity in chemistry." They are, therefore, early introduced in his treatise, and the chapter devoted to their consideration makes one-fourth of the entire volume.

The science of physiology was never more rapidly progressive than it is at the present day, and for this reason we require frequent publications on the subject to keep us informed of the accessions made to it from year to year. The author of the treatise under notice is an original laborer in this field, and is contributing his share towards the advancement of the science. His works are entitled to all the popularity they have enjoyed. His is a vigorous, comprehensive mind; he sees clearly the subject before him, and imparts to his readers a distinct notion of what he considers "the typical facts" of the science, without embarrassing them by details. The subjects of which his several volumes treat possess great attractiveness, and his readers, as they go along with him in the enunciation of his strange and wonderful truths, will find themselves charmed as well as instructed.

